# Digital higher education: a divider or bridge builder? Leadership perspectives on edtech in a COVID-19 reality

**DOI:** 10.1186/s41239-021-00287-6

**Published:** 2021-09-24

**Authors:** Melissa Laufer, Anne Leiser, Bronwen Deacon, Paola Perrin de Brichambaut, Benedikt Fecher, Christian Kobsda, Friedrich Hesse

**Affiliations:** 1 Alexander von Humboldt Institute for Internet and Society, Französische Straße 9, 10117 Berlin, Germany; 2 Global Learning Council/Leibniz Association, Chausseestraße 111, 10115 Berlin, Germany

**Keywords:** Digital learning, Higher education, Edtech, Leadership, Governance, Digital transformation, Inequalities, Access, Collaboration, COVID-19, Mixed methods

## Abstract

The edtech community has promoted claims that digital education enhances access, learning, and collaboration. The COVID-19 pandemic tested these claims like never before, as higher education systems seemingly overnight had to move teaching online. Through a sequential mixed-method approach, we investigated how 85 higher education leaders in 24 countries experienced this rapid digital transformation. Through their experiences, we identified the multiple and overlapping factors that contribute to an institution’s ability to realize the potential of digital education, in terms of access, learning and collaboration, whilst highlighting deeply rooted inequalities at the individual, institutional and system level. Drawing on these empirics, we put forth recommendations for closing the digital divides and pathways forward. Higher education leaders are uniquely positioned to move beyond the emergency adoption of online learning towards inclusive, long-term visions for digital education, which emphasize collaboration over individual gain.

**Supplementary Information:**

The online version contains supplementary material available at 10.1186/s41239-021-00287-6.

## Digitalization of teaching and learning

Optimists have long equated digitization with improving the quality of life and social progress, with the Internet opening up participation in the knowledge society by decentralizing and democratizing information. The same applies to educational technology (edtech), particularly in the discourse driven by edtech providers, which has long heralded learning technologies as means to improve access to education and learning outcomes (Sancho-Gil et al., 2020; Selwyn, 2016). The scholarly debate on edtech effectiveness, however, has been more cautious, and warned of uncritical trends toward digitalization (Castañeda & Selwyn, 2018). Although there is some agreement among scholars that technology may increase access to information, the achievements promised by providers typically fall short (Garcia & Lee, 2020; Mertala, 2020; Selwyn, 2015). Moreover, research on learning effectiveness has been mixed, concluding that tech-focused investment alone cannot make learning better (Bartolomé et al., 2018).

In 2020, the COVID-19 pandemic and the rapid transition to online teaching put edtech promises and research under heightened scrutiny. Prior to COVID-19, fully digitized teaching programs with educational technology embedded across the curriculum were rare and only a few institutions such as open universities had established fully digital models of teaching and learning (Gaebel et al., 2021).

Many believe that the pandemic accelerated the digitalization of higher education and will likely provoke profound and lasting changes (Bozkurt & Sharma, 2020; Fullan et al., 2020). With this study, we aim to explore how these changes contribute to the digital transformation of higher education institutions (HEIs). Specifically, the current study examines how higher education leaders around the world experienced the rapid digital turn, and how edtech promises and claims around access, learning outcomes, and collaboration play out in practice.

### Access

In arguing that digital technologies let individuals retrieve information, use learning materials, and participate in remote learning, edtech providers highlight better access to education. However, ‘access’ is influenced by structural inequalities, which are expressed as geo-demographic variables such as location, income, age, race, or gender (Warschauer, 2004).The term digital divide illustrates the social inequity between individuals who have access to basic infrastructure necessary for digital learning, such as computer devices and the Internet, and individuals who do not (Garcia & Lee, 2020).

A new digital divide presupposes physical access and examines the nature of information technology use. It embodies so-called digital skills (also digital literacy or digital competency) that help learners achieve positive learning outcomes in digital settings but also differ based on level of education, culture, and English skills (Ritzhaupt et al., 2020). As this divide exists between students and teaching staff, HEIs and their faculty may yet be unprepared to adequately foster and develop digital information literacy skills among students (Santos & Serpa, 2017). A digitalization process of teaching must therefore be accompanied by a comprehensive culture change of the learning environment and investment in the digital literacy of stakeholders (Englund et al., 2017; Fischer et al., 2020).

### Learning outcomes

Beyond access, edtech enthusiasts have also claimed that technology improves learning experiences and learning outcomes. For example, in the literature it is argued that learners appreciate digital learning as it enables flexibility, interactivity, and self-pacing (e.g., Hromalik & Koszalka, 2018; Sun et al., 2008). Indeed, research shows that the use of edtech can enhance learning motivation and engagement (Jones, 2020), self-regulated learning (Broadbent et al., 2020), and knowledge transfer (Dohn et al., 2020). Beyond learner enjoyment and cognitive skills, edtech has also shown promise in terms of promoting critical thinking skills, sociocultural learning, student engagement, and learner creativity (Bishop et al., 2020; Lu et al., 2021).

However, researchers have found that several factors mediate the positive learning effects of educational technology. For example, the benefits incurred from digital teaching and learning depend largely on the learning mode, curriculum design, and teaching quality and style (Chen et al., 2018). In addition, for digital learning to be implemented well, instructors need to be equipped with appropriate digital learning pedagogy (see for example Koehler et al., 2013). Lastly, learning is improved when students can choose between different learning modalities and when these offerings fit learners’ needs, the instructional goal, and the nature of the learning task (Chen et al., 2018; Lindberg & Olofsson, 2012). To implement this, a larger culture shift at the institutional level is needed in terms of policy that embraces transformational aspects of digitization and includes careful planning, digital pedagogy, and appropriate tools (Bond et al., 2018; Englund et al., 2017; Fischer et al., 2020).

### Collaboration

In terms of collaborative learning practices, the literature suggests that with the use of edtech, computer-supported collaborative learning (CSCL) can not only enrich the learning experience within and outside of ‘normal’ classrooms (Laurillard, 2009), but in form of virtual collaboration can also introduce intercultural awareness to courses, improve language proficiency, facilitate virtual student mobility, and allow for experiential education experiences (Bruhn, 2016; Júnior & Finardi, 2018). In theory, it can thereby combine global collaboration experiences with local learning and impact (Caniglia et al., 2018). In practice, however, research shows that the collaboration taking place within and between HEIs is often lower than expected (Bond et al., 2018). This finding is replicated for virtual collaboration, where data suggest that “ICT use in curricula and co-curricula is still a low priority in internationalization efforts for HEIs” (Bruhn, 2020, p. 63).

In terms of collaboration and cooperation among teaching staff, we can draw on the concept of open education which Cronin (2017) defines as “collaborative practices that include the creation, use, and reuse of OER [open educational resources], as well as pedagogical practices employing participatory technologies and social networks for interaction, peer-learning, knowledge creation, and empowerment of learners.” (p. 18). She finds that educators are strongly influenced in their decision to use open educational practices by structure and culture. Such findings call for the fostering of collaborative practices at all levels, facilitated by higher education leaders to develop digital literacies and capabilities, inform on privacy and openness, and reflect on the role of higher education institutions in a networked society.

## Study overview

The direct positive relationship between educational technology and ‘better’ education has not been convincingly shown, suggesting that technology is a necessary but not sufficient factor in the digital transformation of education (Castañeda & Selwyn, 2018; Fischer et al., 2020). While the rapid transition to digital teaching in the wake of COVID-19 arguably left institutions little time to reflect on the design and implementation of learning technologies, the unprecedented situation led to widespread adoption of digital tools for teaching. The purpose of this study was thus to understand how the sudden shift measured up against edtech claims, and which factors play a role in the successful implementation of digital teaching and learning.

To test the edtech claims of improving access, learning, and collaboration, we pose the following research question: Does the rapid digital push during the COVID-19 pandemic evoke positive and sustainable development for digital teaching and learning?

To identify the circumstances under which a positive and sustainable development for digital teaching and learning is realized, we pose the following research question: How did higher education leaders experience the opportunities and barriers that arose during the rapid digital turn, specifically related to the edtech promises of access, learning outcomes, and collaboration?

## Research methods

We designed this study as a sequential mixed methods design (Creswell & Plano Clark, 2018), consisting of three data collection rounds: (1) a questionnaire, (2) contextual interviews, and (3) a follow-up questionnaire. The first questionnaire consisted of open and closed questions and explored crisis response and digital learning in early 2020. Based on these, we followed up with in-depth interviews to better understand the contextual features that different institutions and leaders faced during this time. The second questionnaire served to validate these themes in a quantitative manner and at a later point in time, during the fall semester of 2020.

The interview guide and follow-up questionnaire were designed inductively, based on the themes that emerged from the previous rounds of data collection. An integration of methods took place at the stage of designing the instruments of data collection. The three data collections also drew from the same pool of participants, allowing us to collect both in-depth data and explore changes as they occurred over time (Saldaña, 2003).

### Participants

We recruited a diverse sample of higher education leaders working in different types of institutions located in 24 countries (see Additional file 1: Appendix A). Participants were recruited via purposeful and snowball sampling. The participants were informed about the project and the use of their data, before participating in the data collection rounds. Higher education leaders are defined as individuals who possess high-level decision-making capacities in HEIs (e.g., university presidents, deans, directors of digital learning, technology officers, etc.) or in organizations that closely work with universities (e.g., university associations, policy institutions, think tanks, and foundations). This strategic choice enabled us to capture the rapid digital transformation that was occurring within institutions (from a bird’s-eye view) as well as to track digitalization trends as they unfolded across higher education systems. By default, participants were anonymized, however at the end of the first questionnaire participants were given the option of being listed as experts in our study (see Additional file 1: Appendix E).

### Data collection

The first questionnaire ran between May and June 2020. It included questions on the perceived effects of the rapid digital shift on learning outcomes and educational access, asked about the preparedness of the HEI, crisis management responses, greatest challenges in transitioning to online learning, innovations emerging from the pandemic, and expected long-term effects. Because it drew on well-established themes of edtech promises from scholarly literature but also included crisis-specific topics, the questionnaire consisted of a series of open and closed questions (see Additional file 1: Appendix B). In total, we contacted 352 individuals of which 85 participated from 24 countries, resulting in a 24% response rate.

From July to August 2020, we conducted semi-structured interviews (n = 11) with participants from 9 countries. The interviews explored contextual features that emerged in the first questionnaire—digital teaching and learning strategies, pressures the HEI was facing, support it was receiving, challenges and barriers to realizing digital teaching and learning, and collaboration between HEIs (see Additional file 1: Appendix D). The interviews lasted between 26 and 90 min and were recorded, transcribed, and anonymized.

The follow-up questionnaire took place in November 2020 and resulted in 38 responses from 17 countries (from a pool of 62). It investigated whether more strategic approaches to digital education were being pursued in the months following the initial switch to online teaching. Closed-ended questions on challenges mirrored those in the first questionnaire; open-ended questions followed the themes from the interviews, i.e., future visions for digital learning at the HEI, goals of digital teaching and learning strategies, quality of digital teaching, and collaboration between HEIs (see Additional file 1: Appendix C).

### Data analysis

The quantitative data were analyzed using descriptive statistics while the qualitative data were analyzed using MAXQDA. A code book was constructed based on inductive coding (Miles & Huberman, 1994), which was expanded upon to analyze the different data collections. Overall, the data of this study converged, with interview data providing richer accounts and contextually relevant information explaining trends that appeared in the questionnaires. For our findings, we mainly focus on the qualitative data as these provide more context and explanation. In some cases, we draw upon the descriptive statistics of the closed questions from the questionnaires, allowing us to triangulate results from the different collections.

### Findings

Through the experiences of higher education leaders, we address how the rapid digital turn facilitated and hindered access, learning outcomes, and collaboration.

To answer the first research question, we turn to results from the quantitative elements of the first questionnaire. These were largely ambiguous, indicating that further factors played a role in how the rapid digital turn affected digital learning. We then turn to the more nuanced results from the interviews to answer the second research question which elaborated on further factors, providing in-depth accounts of how the rapid change affected students and staff, and the role that institutions and higher education systems play in this process. Findings from the final questionnaire mirrored these findings across all regions and added a future perspective on digital learning.

Because institutions have different starting points, contextual constraints, and demands, there exists no one-fits-all solution around digital learning. We observed variety in how challenges around digital learning manifested, however, found that overarching themes emerged across all contexts. Our data are therefore not clustered by types of HEIs or regions.

Further, we have organized the findings as they relate to the micro (individual experience) and meso (institutions / systems) dimensions, rather than grouping them in accordance with themes in the literature. In reality, access, learning outcomes and collaboration are realized through the actions of individuals and institutions. Focusing on the agency component enables us to illustrate the multiple layers that shape the outcomes of digital education by way of highlighting responses, barriers, and opportunities (see Table 1).

**Table 1:** Findings overview

Dimension	Category	Subcategory	Description
Individual level	Opportunities	Flexible/individualized learning	Digital/remote learning allows for individualized, adaptive learning paths
		Access to education	Digital/remote learning allows for underserved populations to participate in higher education; More lifelong learning opportunities and inclusivity
	Barriers	Infrastructure and devices	Lack of broadband infrastructure and poorly connected regions; Students and teaching staff lack technological devices and/or software
		Home environments	Students’ home environments are described as unsuitable for learning
		Systemic inequalities	Additional hurdles disproportionately affect students from ethnic minorities and/or disadvantaged backgrounds; Some students and staff face additional burdens due to learning/teaching at home (e.g., caring responsibilities, domestic violence, food insecurity, mental health)
		Digital skills, experience, and acceptance	Lack of training amongst students and teaching staff, often due to systemic exclusion from acquiring digital skills; Reluctant attitudes among students and staff toward shift to digital/remote education
Institutional level	Responses	Technical support	Institutions’ actions to close technological and infrastructural gaps by providing students and staff with devices, securing internet access for them, or providing analog alternatives
		Housing	Accommodation provision for students in need
	Opportunities	Cooperation, collaboration and resource sharing	Opportunity for multiple forms of collaboration at various levels, including students, instructors, or HEI leaders cooperating within and between institutions
		Global collaboration and virtual internationalization	Need for global solidarity and collaborative actions between HEIs; Digital higher education can foster new forms of internationalization and student mobility
	Barriers	Systemic inequalities	Inequalities around factors such as race, gender, income, region, etc. are reflected in the HEI system, resulting in the uneven distribution of resources within and between regions and countries
		Governmental support	Amount and type of governmental support and funding that HEIs are given for digitalization vary greatly

### Individual-level opportunities

Several respondents across all data collections positively linked the shift to digital education to flexible and individualized learning. The individualized/personalized learning model focuses on increasing learning flexibility by tailoring teaching and learning practices to fit students’ needs, capabilities, preferences, and constraints (Wanner & Palmer, 2015). Individualized learning was often conceptualized as part of a mixed-model, as described by a higher education leader working in the Asia–Pacific region^1^:

“Personalized [learning] […] would give them [students] more freedom of not coming to the physical venue so that they could review the material on their own time and at their own place. But it would not be a hundred percent because the students want a mixture.”

Beyond sparking shifts in teaching and learning practices, many respondents associated digital education with lifelong learning possibilities and increased access to education for underserved populations. Indeed, the growing diversity of digital formats multiply the uses and potential users of lifelong learning opportunities. The flexible configuration of digital higher education can be attractive to a range of persons unable to abide by the constraints of traditional in-person teaching. A higher education leader located in the United Kingdom summarized how digital alternatives can make higher education more inclusive:

“I do think that there is an equalizer, there is a leveling effect. And that leveling effect is that, whereas it might have been more difficult for some people to travel to a university because they couldn't afford it, they can't give up the job that they've got, they can't afford the transport. They have caring responsibilities, so they can't move away from home. [Digital education] actually opens up access for those people, provided that you can afford the equipment and the technology, and you know how to use it.”

The access issue is particularly poignant when taking into account the unequal distribution of caring responsibilities, which are still overwhelmingly ascribed to women (Charmes, 2019). A respondent from South Africa recounted how digital higher education could change one of their student’s life:

“What she [the student] did this year was she simply deregistered from her master's, to help her mother who'd had another baby, because that is the role of women. [...] Digital consultations will change her future. They really will...”

In addition, digital education tools could help even the playing field within the student body by offering extra resources. A higher education expert based in the United States underlined the correlation between race, ethnicity, and income, arguing that digital learning tools could bridge the digital divide between students:

“This issue of race and ethnicity [is] very, very different in the U.S. But almost all of that is economically correlated with income. [...] The digital learning tools that we can use in a classroom often support those [students], that haven't had the same kind of practice, [compared to] those that come from high-income groups.”

Our findings reflected optimistic attitudes toward digital education and the opportunities it offers. Participants described the ways in which digital education can improve teaching and learning by tailoring it to individuals’ needs, and by increasing access to higher education for many previously excluded parties. However, they did not do so uncritically, especially when digital formats’ radical potentials came into sharp contrast with unequal realities.

### Individual-level barriers

The above-listed opportunities brought about by digital teaching existed in tension with various barriers, which prevented individuals from participating in online teaching and learning formats. Claims that digital education can even the playing field between students rely on the premise of staff and students having stable access to infrastructure and devices, which has proven to be fragile in the sudden move to remote teaching. In turn, when this access cannot be secured, digital education can heighten already existing inequalities between students, disproportionately affecting students from ethnic minority and disadvantaged backgrounds.

Throughout the questionnaires^2^ and interviews, the majority of respondents from both the Global North and South listed students’ lack of access to computers or to a stable Internet connection as the main obstacles in the rapid shift to online learning. Many were not aware that students had been relying entirely on the universities’ resources, as described by a participant working in a German university:

“It was rather surprising to hear that my conception that I had that basically every student that is studying here is equipped with a laptop is not the case. So, there were differences, and these differences form of course bottlenecks in regard to accessibility.”

The crisis brought forward vast inequalities within student bodies, reinforcing them by removing the equalizing space of the university campus. These inequalities were sometimes linked to students’ homes being in poorly connected regions, reported in a similar fashion by higher education leaders in Kenya:

“She walks 2 kilometers from her home every day, sits there in the church and does her homework, attends class, does everything because outside of that place [there] is no Wi-Fi.”

And in the United Kingdom:

“Even though we pride ourselves on kind of being a digital nation, if you like, we know that there's [...] coastal and rural areas where broadband is just a dream. [...] It will exacerbate those existing inequalities unless we do something about the infrastructure.”

Furthermore, students’ home environments not only often lacked the technological equipment, but they were also not always conducive to learning. Faculty noted how many students shared spaces with their families and did not have quiet environments to study. These factors reinforced a digital divide between students, as described by a respondent:

“In the short term, digital learning is likely to lead to inequity in access to learning due to the digital divide that also relates to economic inequality and geography. Once these factors are addressed at the national level, equity in education will return.”

Barriers around systemic inequalities were also present for many students. Having previously pointed to the ways in which online learning could improve access to higher education for women by lending them more flexibility, it is important to note that women were disproportionately affected in a myriad of ways by teleworking and online studying. Levels of domestic violence have risen sharply around the globe over the past year (UN Women, 2020), and closed university campuses meant returning to less safe home environments for many women, as a participant from South Africa explained:

“Many students come from a background of poverty, sometimes extreme poverty. And I know that their family situations are very, very difficult. You know that we have a high level of violence. [...] We have the highest level of femicide. [...] The university is, I was going to say violently opposed to the whole situation with women as an attempt to make the campus as safe as possible. That safety has gone away with people working at home.”

The same higher education leader from South Africa also noted that even with the increased flexibility of online learning, women are often overburdened with caring responsibilities at home:

“Family responsibilities for everyone at the moment tend to overwhelm their activities online. And we do see evidence internationally that women are more affected.”

Digital divides are exacerbated along the lines of gender, race, location, and economic background (Warschauer, 2004). This holds true in our data among both students and staff, and encompasses differing access to online learning, structural exclusion from the digital realm, and divergences in digital skills. Moreover, the first questionnaire indicated that respondents were pessimistic about digital education increasing the access for disadvantaged groups, with circa 58% of respondents either disagreed or were neutral regarding digital education promoting inclusivity.

In addition, the sudden shift to online teaching proved challenging as staff had varying levels of digital skills, experience, and acceptance. In the questionnaires, respondents reported that “lack of expertise regarding remote/online teaching among university instructors” was the biggest challenge their institution faced. This indicates that digital literacy among teaching staff continues to be a major hurdle for institutions—one they had not been able to fully solve nine months into the pandemic.

Furthermore, respondents reported reluctance among faculty to adopt online teaching. This disinclination was perceived as a barrier to reaping the potential benefits of digital education and making the most of the opportunities it presents. One participant linked this attitude among staff to a generational digital divide:

“Although the university administration is trying to rally the faculty around remote learning the digital divide between younger and older faculty is too wide and as a result, unions tend to favor the views of the older faculty.”

The barriers for the successful implementation of digital higher education thus encompass a wide range of factors, from digital divides among staff caused by lack of training, reluctant attitudes, or systemic exclusion, to inequalities among the student body which are heightened by and/or come about under the COVID-19 crisis.

### Institutional-level responses

The findings also revealed creative solutions universities undertook to address inequalities amongst their students and staff, first and foremost by providing technical support. For example, participants from nearly all world regions described institutional programs that were quickly implemented to provide laptops to disadvantaged groups, a group that ranged anywhere from 15 to 80% of the student population. In the quote below, a respondent shares their institution’s approach for addressing inequalities:

“About 15% of our students did not have access to computers or other devices. As a result, we purchased 5,000 computers and established a computer loan facility to provide devices to these students ...”

Institutions also sought different strategies to overcome Internet connectivity problems. These ranged from setting up Internet ‘hot-spots’ in parking lots to giving students USB modems. In several cases, institutions formed partnerships with private companies to ensure Internet access for their students and staff. A higher education leader from Kenya describes such a partnership:

“[the university network] teamed up with a communication company [...] which gave us sim cards with 10 giga per month. That was made available for our lecturers [...] because a number of them don’t have reliable Wi-Fi at home."

However, it was not always possible to solve access issues and institutions had to turn to alternative strategies to reach their students. For instance, one respondent described how their institution provided hard copies and telephone tutoring for students who did not have Internet access, as well as for international students who had already left the country. In another example, a respondent from South Africa describes how access issues were considered when making *housing* arrangements:

“Some students were still challenged as their living circumstances did not enable learning. We have now addressed this by allowing a return of those students to residences so that they could undertake online learning from their residences.”

Many institutions’ initial crisis response focused on addressing these immediate access challenges, however increasingly the topic of collaboration emerged, and how digitalization may be harnessed to grow collaboration among and across institutions.

### Institutional-level opportunities

Manifesting differently across the data, collaboration was considered, by the majority, essential to the digitalization process and in some cases even key to realizing its full fruition. In the second questionnaire, the majority of participants (66%) agreed with the findings from the interviews, that cooperation, collaboration and resource sharing was a necessary step to ensure students’ equal access to higher education. This sentiment is shared by a respondent from South Africa:

“The value [of collaboration] is knowledge and skills sharing, deepening peer interaction including evaluation, and disrupting knowledge silos. Developing common courses and co-teaching […] will expand expertise and access for students.”

In some cases, these collaborations between universities were based on geographical proximity as reported by a participant from the Asia–Pacific region:

“We have found it very helpful to collaborate nationally with universities, more on approaches and rapid shared learnings rather than resources.”

Other respondents highlighted the need for global collaboration; as one participant put it: “we are all in the same boat”. A respondent from a university association confirmed this phenomenon, describing how higher education associations had become “a sort of a rallying point” for institutions looking for answers and support. Along these lines, a participant from South Africa pointed out how the current crisis showed that global solidarity among HEIs is needed to address future challenges:

“Pandemics cannot be addressed unless we have quality institutions globally to address them. This means that we are increasingly going to have to build equitable networks of universities so that we can respond to the transnational challenges of our time.”

This global dimension of collaboration also resonated with a discussion about bridging inequalities between the Global North and South. In the following, a higher education leader from the United States describes the actions that need to be undertaken:

“I think that collaboration is absolutely essential if we are to meet the challenges of this historical moment. All of our challenges are transnational in character, and we require institutional capacities and human capabilities to address this across the world. This means that we need to reimagine our global collaboration to teach across institutions, nations, and continents.”

The respondent went on to caution that existing university partnerships prevent collaborative approaches to countering education challenges, instead such partnerships often contribute to brain drain as “universities are too focused on brand[ing] that they have forgotten their institutional mission.” This criticism was echoed by a South African participant who pointed to the international student recruitment strategy pursued by universities in Western countries:

“As of now institutions cross-subsidize their domestic students with international students, many of whom come from the developing world. This is done in the name of global solidarity and makes a mockery of collaborative notions of partnerships.“

Related to this topic was virtual internationalization, which was seen to be a more equal approach to mobility. Virtual mobility could even the playing field as it opens opportunities for students who may not have been able to afford to participate in traditional international mobility schemes; a respondent from the Asia–Pacific region described how students from lower income economies had been engaging with their offered online activities at a high rate.

This new type of online mobility may, however, bring about the reduction of physical international exchanges and thus destabilize institutional business models built around the flow of international students. This could upend income generated by international students, which contribute to financing a university’s endeavors.

In the data, collaboration took on different forms and appeared to be an ideal towards which institutions were striving, however in some instances it took a backseat, as we describe below, due to more pressing resource issues.

### Institutional-level barriers

Several barriers were identified that prevented institutions from fully realizing digital education and collaboration. One respondent from the Asia–Pacific region explained collaboration was not a priority as “it was enough of a struggle to get our own house in order,” while another participant from the United Kingdom remarked that heightened competition within the higher education system had made institutions distrustful of collaborating. Another explanation offered by a participant from the United States was that institutions within the higher education system were simply too diverse to find common ground for collaboration.

These explanations hint at another prominent element in the data, namely the deep gulfs of systemic inequalities when it comes to the distribution of resources between institutions and within systems. For example, several participants remarked that smaller institutions or those geared towards teaching or serving underrepresented populations, were hit especially hard with the rapid digitization, as they had fewer resources available to them compared to large, ‘elite’ research universities. In the following, a higher education leader from the United States reflects on such embedded inequalities:

“... our historically black community colleges […] have been really reliant on a physical campus, where the strong learning community is how their students are successful […] and to shift to digital [education] has been extremely hard, because they don't have the people, they don't have the infrastructure. And that means they don't have the investment capability in those things.”

In addition, institutions faced financial challenges, struggling to purchase much needed licenses and software. A respondent from Kenya described how their institution had to shoulder an additional financial burden due to the pandemic and its impact on the local economy, as parents’ inability to pay their children’s fees made the institution’s fee revenue go down almost 50 percent.

Furthermore, several respondents commented on how governmental support was not robust enough to allow institutions to digitalize, even when the desire was there, as illustrated in the quote from a leader of a German university:

“In regard to digitalization as a whole, I think we were from an economic standpoint doing the minimal because to be honest the money that we received from the government or from the Senate [for] digitalization measures is still based on a more linear conception that is stemming from the 90s and 2000s.”

While some respondents noted that other income streams could cover funding gaps such as through sport programs, donations, and endowments, these options were by no means a universal reality for all institutions. Moreover, in some cases governmental funding was actually being cut due to the crisis, as described by a higher education expert in South Africa:

“All government departments have had a 20% cut, and that includes the Department of Science and Innovation, with which I work quite closely. They have had to decide how to distribute that cut. It includes the Department of Higher Education and Training from which the major income comes to the universities. It is the biggest funder of research in the country. [...] My colleagues tell me that many South African universities will be facing bankruptcy.”

The respondent explained that these budget cuts were in addition to others that had happened in recent years, emphasizing the weight of this additional financial burden.

The findings highlight the various ways in which digital education may be realized, its positive and challenging consequences for individuals and institutions. COVID-19 and the rapid digitalization held a mirror up to digital education, allowing us to empirically investigate the rationales in the literature and edtech discourse.

## Discussion and conclusions

Tech evangelists have long linked digitization to societal improvement, citing how the Internet decentralizes and democratizes information and participation in the knowledge society; sentiments which are also echoed within the edtech discourse (Dron & Anderson, 2014). In this study, we investigated how higher education leaders around the world experienced the rapid digital turn and the outcomes associated with digital education. Especially among edtech providers, technology has been frequently heralded as means to improve access, learning outcomes, and collaborative practices (Sancho-Gil et al., 2020; Selwyn, 2016). On the other hand, scholarly debate on this topic is more nuanced, highlighting inequalities related to the digital divide (unequal access to technical resources) (Garcia & Lee, 2020; Warschauer, 2004) and the new digital divide (differing levels of digital skills) (Ritzhaupt et al., 2020). In addition, researchers caution that implementing digital learning is not simply a switch to online formats, but requires strategy and leadership specifically geared at implementing technology-enhanced learning and digital transformation of the HEI (Arnold & Sangrà, 2018).

The scholarly literature hints at the complexity involved in implementing educational technology for learners, instructors, and institutions. Our inductive themes further build on this, highlighting the many facets of this complexity, and expanding on the existing themes of access, learning outcomes, and collaboration. At first glance, several of the positive claims made by edtech providers and enthusiasts resonated with the experiences of the respondents, with many reporting on the new opportunities digital education offers to students, such as providing individualized learning experiences and lifelong learning opportunities. In addition, arguments were made regarding the ‘equalizing effect’ of digital education, with examples given of how it eased access to higher education for non-traditional students and underrepresented groups. Yet, in the same breath, the findings displayed numerous examples of digital divides between students, within the teaching staff and across institutions. For instance, some respondents explained that university campuses not only provide basic infrastructure for students to carry out their studies, but also a safe haven from violence and home environments not conducive for learning. These inequalities spanned across all regions and were often drawn across existing divisions in societies, related to individual economic status, gender, race as well as a country’s historical context, distribution of wealth and the economic system.

These contradictions in the promises and realities of digital education were largely acknowledged by the respondents, and many sought to address them to the best of their ability. For example, many institutions turned to creative solutions to quickly remedy the lack of technical resources among their students and staff. These ranged from setting up laptop loaning schemes to creating Internet hot spots in parking lots. In some cases, collaborations were born as more long-term solutions to tackling inequalities and challenges related to the rapid digital transition. These collaborations often built upon existing networks, such as university associations, which became ‘rallying points’ during the crisis. A desire for solidarity was often a driving force behind such collaboration, however inequalities again became a barrier. To collaborate, institutions first needed access to basic infrastructure, or as one higher education leader explained, “it was enough of a struggle to get our own house in order”. Thus, institutions that held precarious positions within their own systems—due to their size, emphasis on teaching, serving underrepresented groups, or limited governmental funding—were doubly burdened in implementing digital teaching and therefore less able to engage in collaboration.

Our findings indicate that the positive digital education outcomes attributed to edtech (see Bishop et al., 2020) are more than just pipe dreams and do in part reflect reality. However, before these outcomes can begin to be realized, we must first be aware of and address the fundamental inequalities that prevent participation in digital learning. Similarly, collaboration, arguably the emergent and untapped outcome of edtech, is also dependent on digital literacy, or lack thereof, among all university stakeholders (Cronin, 2017). Moving forward, a new paradigm of collaboration might encompass collaborative learning practices (Laurillard, 2009), global collaboration in terms of internationalization efforts (Leask, 2013), and cooperation around resource sharing and open pedagogy (Bali et al., 2020). Strong leadership and a strategic and inclusive approach are needed, lest educational technology becomes another empty promise among other quick-fix ideas.

We acknowledge the limitations of our study. First, our sample is not representative of all diversity that spans across the higher education landscape. Despite this limitation, our findings indicated shared themes across regions, thus demonstrating that many institutions are facing similar challenges, albeit with some contextual differences. Second, the focus on higher education leaders limited our insight into the experiences of instructors and students. We recommend that future research further address macro (e.g., national policies addressing inequalities) and micro (e.g., students’ digital learning experiences) dimensions to understand how these levels interact.

In conclusion, we recommend that collaboration be understood as encompassing collaborative learning, internationalization, and collaboration/cooperation between HEIs. Such a comprehensive understanding of collaboration goes well beyond a pedagogical focus, instead enabling transformational change of the higher education system and redressing injustice (see also Bali et al., 2020). To facilitate policies and programs around digital learning and collaboration on an institutional, national, and international level, we also recommend a scholarly and practical focus on digital education leadership or e-leadership (see Arnold & Sangrá, 2018). Higher education leaders, especially those working in well-funded institutions, are uniquely positioned to spearhead collaborations in the digital sphere and build bridges across the gulfs of inequalities: by sharing their resources with other individuals and institutions, pushing the rhetoric about collaboration over individual gain, and creating awareness both internally and externally of hidden inequalities that can be tackled using digital technologies. Moreover, existing university networks can be expanded upon and new constellations constructed, as one participant succinctly put it, “pandemics cannot be addressed unless we have quality institutions globally to address them.” The future will likely hold new global challenges that rely on international collaboration across the global knowledge community.

## Supplementary Information

**Additional file 1: Appendix A** Participant overview. **Appendix B** First questionnaire. **Appendix C** Second questionnaire. **Appendix D** Interview guide. **Appendix E** List of experts.

## Data Availability

The datasets generated and/or analyzed during the current study are not publicly available due to anonymity terms established with the research participants but are available from the corresponding author on reasonable request.

## Footnotes

1 We have adopted the regional term “Asia–Pacific” for some participants due to anonymity reasons.

2 In the first questionnaire, “lack of technical resources (e.g., Internet access, computers, other equipment)” was ranked the 2nd biggest challenge (out of 8 options) among respondents. In the second questionnaire, “lack of technical resources” was ranked as the 3rd biggest challenge among the respondents.
