# Supplementary material for: Digital higher education: a divider or bridge builder? Leadership perspectives on edtech in a COVID-19 reality
Source: Int J Educ Technol High Educ. 2021 Sep 24;18(1):51. doi: 10.1186/s41239-021-00287-6 (PMC8460315; doi:10.1186/s41239-021-00287-6)
Supplement: Supplementary file 1 — Additional file 1: Appendix A Participant overview. Appendix B First questionnaire. Appendix C Second questionnaire. Appendix D Interview guide. Appendix E List of experts. [file 41239_2021_287_MOESM1_ESM.docx]

Appendix A

Participant Overview

| **Institution of Participants** | |
| --- | --- |
| Leaders at Higher Education Institutions | 78 |
| Leaders at Intermediaries | 9 |
| **Countries** | |
| Australia | 3 |
| Austria | 4 |
| Belgium | 2 |
| Brazil | 5 |
| Canada | 2 |
| China | 1 |
| Denmark | 2 |
| Egypt | 1 |
| Finland | 1 |
| Germany | 9 |
| Ghana | 2 |
| Hong Kong | 3 |
| Kenya | 6 |
| Mexico | 2 |
| Netherlands | 4 |
| Nigeria | 1 |
| Russia | 1 |
| Singapore | 5 |
| South Africa | 6 |
| Sweden | 2 |
| Switzerland | 5 |
| United Kingdom | 8 |
| United States | 8 |
| Vietnam | 1 |
| Unknown | 1 |
| **Size of Higher Education Institution** | |
| Less than 1,000 students | 1 |
| Less than 5,00 students | 4 |
| 5,000–10,000 students | 5 |
| 10,000–20,000 students | 10 |
| 20,000–50,000 students | 36 |
| 50,000–100,000 students | 15 |
| More than 100,000 students | 5 |
| **Type of Higher Education Institution^[[1]](#footnote-1)^** | |
| Applied university | 5 |
| Technical university | 3 |
| Public university | 30 |
| Public (research university) | 17 |
| Research university | 2 |
| Private university | 10 |
| Music & arts institution | 2 |
| Open university | 3 |

Appendix B

First Questionnaire

Thought Leader Questionnaire: Higher Education during COVID-19

The COVID-19 pandemic has brought rapid changes to the higher education landscape. In this thought leader questionnaire, the Global Learning Council and its partners aim to capture how universities and related higher education organizations are coping with the current situation. Specifically, decision-makers and thought leaders working in universities and organizations that support universities are invited to participate.

The questionnaire is part of a two-step study. The second step will build upon the results of this questionnaire. Participants interested in partaking in the second step are asked to provide their contact information in the last section. We encourage participants to circulate the questionnaire among their contacts. The questionnaire will take approximately 10 minutes to complete.

| Where do you currently work? *  Please choose **only one** of the following:  ⃞ At a university / higher education institution  ⃞ At an organization that works with universities  ⃞ Other |
| --- |

| Please specify your current job title or position within your organization. *  Please write your answer here: |
| --- |

| Please tick the boxes that apply to your university. *  Only answer this question if the following conditions are met:  Answer was 'At a university / higher education institution ' at question '1 [Q1]' (Where do you currently work?)  Check all that apply  Please choose **all** that apply:  ⃞ Public university  ⃞ Private university  ⃞ Research university  ⃞ Applied university  ⃞ Technical university  ⃞ Online university  ⃞ Open university  ⃞ Arts & music institution  ⃞ Other: |
| --- |

| Please tick the boxes that apply to your organization. *  Only answer this question if the following conditions are met:  Answer was 'At an organization that works with universities ' *or* 'Other' at question '1 [Q1]' (Where do you currently work?)  Check all that apply  Please choose **all** that apply:  ⃞ Nonprofit  ⃞ For-profit  ⃞ Governmental  ⃞ Non-governmental  ⃞ Research institution  ⃞ Think tank  ⃞ Foundation  ⃞ Policy institution  ⃞ University association  ⃞ Regional  ⃞ National  ⃞ International  ⃞ Supranational  ⃞ Other: |
| --- |

| How many students does your university / higher education institution have? *  Only answer this question if the following conditions are met:  Answer was 'At a university / higher education institution ' at question '1 [Q1]' (Where do you currently work? )  Please choose **only one** of the following:  ⃞ Less than 1,000 students  ⃞ Less than 5,000 students  ⃞ Between 5,000 - 10,000 students  ⃞ Between 10,000 - 20,000 students  ⃞ Between 20,000 - 50,000 students  ⃞ Between 50,000 - 100,000 students  ⃞ More than 100,000 students |
| --- |

| In what country is your organization located? *  Please write your answer here: |
| --- |

| In the following statements, we would like you to assess the impact of the COVID-19 pandemic on higher education. Please indicate to which degree you agree or disagree with the statements. * [closed, Likert scale]  [Scale: 1, disagree completely -- 5, agree completely, does not apply]  Only answer this question if the following conditions are met:  Answer was 'At a university / higher education institution ' at question '1 [Q1]' (Where do you currently work?)  Please choose the appropriate response for each item:   1. **Politicians in my country are able to support higher education institutions during the COVID-19 pandemic.** 2. **My university is struggling to respond to the COVID-19 pandemic.** 3. **Prior to COVID-19, my university was prepared for digital teaching.** 4. **During the COVID-19 pandemic, it is important to relax the rules and regulations of teaching in higher education institutions.** 5. **The rapid shift to remote / online teaching makes the digital divide among faculty members more apparent.** 6. **The rapid shift to remote / online teaching will lead to poorer learning outcomes than prior to the pandemic.** |
| --- |

| In the following statements, we would like you to assess the impact of the COVID-19 pandemic on higher education. Please indicate to which degree you agree or disagree with the statements. *  [Scale: 1, disagree completely -- 5, agree completely, does not apply]  Only answer this question if the following conditions are met:  Answer was 'At an organization that works with universities ' *or* 'Other' at question '1 [Q1]' (Where do you currently work?) Please choose the appropriate response for each item:   1. **Politicians in my country are able to support higher education institutions during the COVID-19 pandemic.** 2. **In the country where I work, universities were prepared for digital teaching prior to COVID-19.** 3. **During the COVID-19 pandemic, it is important to relax the rules and regulations of teaching in higher education institutions.** 4. **The rapid shift to remote / online learning makes the digital divide among faculty members more apparent.** 5. **The rapid shift to remote / online learning will lead to poorer learning outcomes than prior to the pandemic.** |
| --- |

| What are the greatest challenges in transitioning to remote / online learning for universities in response to the COVID-19 pandemic?  Please note: The following list is based on the experiences of the project members and may be incomplete. Please complete the list if you feel that an important challenge is missing. *  Please select from 1 to 3 answers.  ⃞ Lack of technical resources (e.g., internet access, computers, other equipment)  ⃞ Lack of digital infrastructure at the university  ⃞ Lack of expertise regarding remote / online teaching among university instructors  ⃞ Lack of motivation among university instructors  ⃞ Lack of motivation among students  ⃞ Lack of technical staff at universities  ⃞ Lack of institutional funding  ⃞ Difficulties in formulating a collective institutional strategy  ⃞ Does not apply  ⃞ Other: |
| --- |

| From your perspective, what are the specific challenges your university is currently facing when it comes to organizing teaching? What measures is your organization undertaking to meet these challenges? *  Only answer this question if the following conditions are met:  Answer was 'At a university / higher education institution ' at question '1 [Q1]' (Where do you currently work?)  Please write your answer here: |
| --- |

| From your perspective, what are the specific challenges universities are currently facing when it comes to organizing teaching? What measures is your organization undertaking to meet these challenges? *  Only answer this question if the following conditions are met:  Answer was 'Other' *or* 'At an organization that works with universities ' at question '1 [Q1]' (Where do you currently work?) Please write your answer here: |
| --- |

| In some respects, the COVID-19 pandemic is forcing us to take new paths in university teaching. Is there a technical or social innovation that surprised you positively? Please explain why. *  Please write your answer here: |
| --- |

| In the following statements, we would like you to assess the long-term effects of the COVID-19 pandemic on higher education. Please indicate to which degree you agree or disagree to the following statements. ** [closed, Likert scale]  [Scale: 1, disagree completely -- 5, agree completely]  Please choose the appropriate response for each item:   1. **The COVID-19 pandemic will ultimately lead to more digitized teaching.** 2. **The push for digital education will make higher education more inclusive and accessible for socially disadvantaged students.** |
| --- |

| In your perspective, how do you think the COVID-19 pandemic will affect higher education in the long term? *  Please write your answer here: |
| --- |

| May we list your name and affiliation in future publications? With this allowance, we will not link your name to the answers you provided. If yes, please provide us with your name and organization. |
| --- |

| This questionnaire is part of a two-step study. The anonymized results of the first questionnaire will be shared with participants who choose to partake in the second step. Would you be interested in participating in a second step in the near future?  If yes, please provide us with your email address in the text box below.  You are also welcome to get in touch with us via email if you would like to be informed about the results without taking part in the second step. |
| --- |

Appendix C

Second Questionnaire

Thought Leader Questionnaire: Mapping the New Normal for Higher Education

We would like to thank you for your earlier participation in this research project. We would now like to invite you to participate in the last step of our study - Leading in Times of Crisis: Higher Education Leadership during the COVID-19 Pandemic. This questionnaire builds on the results of our first questionnaire (“Thought Leader Questionnaire: Higher Education during COVID-19), which you participated in last May - June.

Our study focuses on how higher education leaders - working in higher education institutions and intermediaries around the world - are coping with the rapid transition to online / remote teaching and learning. In this questionnaire, you will be given the opportunity to reflect on the outcome of the first questionnaire as well as space to share experiences from your institutional and higher education context. The questionnaire consists of a series of closed and open questions. It will take approximately 15 minutes to complete.

| Please enter your name. *  Please write your answer here:  This information is for internal use only and allows us to match your responses to those you entered in our previous questionnaire, which you participated in last May - June. |
| --- |

| Please enter the name of your organization. *  Please write your answer here:  This information is for internal use only and allows us to match your responses to those you entered in our previous questionnaire, which you participated in last May - June. |
| --- |

| Where do you currently work? *  Please choose only one of the following:  ⃞ At a university / higher education institution  ⃞ At an organization that works with universities  ⃞ Other |
| --- |

| In the first questionnaire, we asked respondents to assess the main challenges universities were facing while transitioning to online / remote teaching in response to the COVID-19 pandemic.  In your opinion, which of these challenges are universities currently facing with digital teaching and learning? Please select the top three. * Please choose all that apply:  ⃞ Lack of technical resources (e.g. internet access, computers, other equipment)  ⃞ Lack of digital infrastructure at the university  ⃞ Lack of expertise regarding remote / online teaching among university instructors  ⃞ Lack of motivation among university instructors  ⃞ Lack of motivation among students  ⃞ Lack of technical staff at universities  ⃞ Lack of institutional funding  ⃞ Difficulties in formulating a collective institutional strategy  ⃞ Does not apply  ⃞ Other: |
| --- |

| A few more challenges came to light over the course of our study. In your opinion, which of these challenges are universities currently facing with digital teaching and learning? Please select the top three. *  Please choose all that apply:  ⃞ Lack of digital infrastructure in the country  ⃞ Lack of digital infrastructure in a student’s home environment  ⃞ Lack of experience among students in self-directed learning  ⃞ Lack or limited transferability of study content to online formats  ⃞ Lack or limited understanding of how digital teaching modalities impact learning  ⃞ Difficulties technically implementing online examinations (e.g., remote proctoring)  ⃞ Difficulties assessing skills in online examinations (e.g., practical skills)  ⃞ Increased workload among instructors  ⃞ Does not apply  ⃞ Other: |
| --- |

| A key element for moving towards long-term digital teaching and learning is setting up evaluation mechanisms to ensure the quality of digital teaching.  Among institutions in your network, what measures are being taken to ensure the quality of digital teaching? *  Please write your answer here:  *Evaluation mechanisms may include monitoring learning outcomes, teaching performance, setting up benchmarking, teaching & learning analytics, etc. |
| --- |

| The rapid transition to digital teaching and learning provided the opportunity for many to experiment with different teaching modalities.  What digital teaching modalities do you believe are best suited for your student body and can enhance their learning experience? *  Please write your answer here:  *Teaching modalities may include blended learning, personalized learning, adaptive learning, synchronous & asynchronous online learning, etc. |
| --- |

| The rapid transition to digital teaching and learning provided the opportunity for many to experiment with different teaching modalities.  Among institutions in your network, what digital teaching modalities are being pursued to enhance the student learning experience? *  Please write your answer here:  *Teaching modalities may include blended learning, personalized learning, adaptive learning, synchronous & asynchronous online learning, etc. |
| --- |

| Many institutions will need to adapt their digital teaching and learning strategies in order to remain competitive in the future.  What goals do you think your institution should emphasize in their digital teaching and learning strategy? *  Please write your answer here:  *A digital teaching and learning strategy may include drawing from different recruitment pools (non-traditional, international, & regional students), setting-up certificate programs, and private-public partnerships, which involve a higher education institution cooperating with the private sector. |
| --- |

| Many institutions will need to adapt their digital teaching and learning strategies in order to remain competitive in the future.  Among institutions in your network, what goals do you think should be emphasized in their digital teaching and learning strategies? *  Please write your answer here:  *A digital teaching and learning strategy may include drawing from different recruitment pools (non-traditional, international, & regional students), setting-up certificate programs, and private-public partnerships, which involve a higher education institution cooperating with the private sector. |
| --- |

| During the pandemic, some universities collaborated closely with other universities or university associations.  What value do you see in collaborating with other universities, on a national or international level? What would you like to see your institution collaborate on? *  Please write your answer here:  *Collaboration may include sharing expertise, best practices, open educational resources, and building knowledge and research exchanges. |
| --- |

| During the pandemic, some universities collaborated closely with other universities or university associations.  What value do you see in collaborating with other universities, on a national or international level? What would you like to see your institution in your network collaborate on? *  Please write your answer here:  *Collaboration may include sharing expertise, best practices, open educational resources, and building knowledge and research exchanges. |
| --- |

| In the following statements, we would like you to assess the long-term effects of the rapid transition to online / remote teaching and learning. * Please indicate to which degree you agree or disagree with the statements. * [closed, Likert scale]  [Scale: 1, disagree completely -- 5, agree completely, does not apply]   1. **If universities are able to deliver high-quality digital teaching beyond the COVID-19 pandemic, it will give**   **them a strategic edge over competitors**.   1. **In the future, teaching will become a more central mission for universities than in the past.** 2. **Students’ access to higher education can only become more equitable if universities share their resources and collaborate with other universities.** 3. **Quality teaching in the future will depend on a balanced mixture of teaching modalities.** 4. **In the future, we will see more public private partnerships in higher education.** 5. **Once we perfect student engagement and community-building through digital tools, campuses will no longer be necessary for the higher education experience.** |
| --- |

| Do you have any further thoughts on digital teaching and learning? Please write your answer here: |
| --- |

Appendix D

Interview Guide

Prior to COVID-19

- (HEIs) - Prior to COVID-19, did your institution have a digital teaching strategy in place? Can you tell me about it?
  - *Follow-up questions:* When was it developed? How concrete was it? Who was involved in the strategy? Was it already being implemented? How do you think your institution compares to other HEIs?
- (ORGs) - Generally speaking, what was the approach HEIs in your system (country) had towards digital teaching and learning prior to COVID-19? Can you tell me about it?
  - *Follow-up questions:* Did institutions have digital teaching and learning strategies? How do you think the approach to digital teaching in your system compared to other higher education systems?

Digital Teaching & Learning

- During COVID-19, online teaching and learning was often seen as a solution to continuing teaching while schools and universities were closed. Do you feel that your university / higher education system has moved beyond this emergency response? What would you name the phase you are in right now?
- What are the current goals driving your institution / system to continue digital teaching and learning?
  - *Examples:* Improve quality of teaching; Make teaching more individualized; Make teaching more effective; Make teaching more inclusive; Expand the student body (e.g., offer executive education or lifelong learning)
  - What is the end goal (five years in the future)?

Contextual Features

- What were the economic rationales (e.g., revenue generation, reaching different student populations) driving digital teaching and learning? Has this changed since COVID-19?
- Did HEIs receive political support during COVID-19? What did that support look like? Were there other kinds of political support you would have hoped for?
- How much autonomy do universities in your context have to set their own digital teaching and learning agendas?
- Reflecting on the students in your higher education system, did the crisis expose existing or unexpected  inequalities? Does being aware of these inequalities shape the digital teaching approach taken by universities?
- Do you feel your HEI / HEIs in your system have the necessary resources to continue digital teaching? What is the largest resource challenge faced by your institution / HEIs in your system?
  - *Examples:* Broadband and electricity; technical infrastructure; pedagogical skills (Faculty training; digital skills (Faculty training); educational resources
- Reflecting on your own context, what structural features in your institution / system support digitalization? Or hinder digitalization?
  - *Examples:* institution’s decentralized structure, its size, the composition of the student body, type of teaching, etc.
- Did your university / universities turn to external organizations, such as university networks and associations or others, for support? What did this support look like and how did it turn out?
- Did you observe that one or a handful of universities were being positioned as best case examples?

Appendix E

List of Experts

- Andrea Schaub - Universität Tübingen
- Carl Wieman - Stanford University
- Tim van der Hagen - Delft University of Technology
- Martin Vetterli - École Polytechnique Fédérale de Lausanne
- Arvid Kappas - Jacobs University Bremen
- Sylvain Charbonneau - University of Ottawa
- Walkyria Magno e Silva - Universidade Federal do Pará
- David Cardwell - University of Cambridge
- Barbara Lee - Rutgers University
- Nana Aba Aappiah Amfo - University of Ghana
- Liz Marr - The Open University
- Martin Veller - University of the Witwatersrand
- Izael Pereira Da Silva - Strathmore University
- Geert ten Dam - University of Amsterdam
- Tawana Kupe - University of Pretoria
- Karen Vignare - Association of Public & Land Grant Universities
- Christiane Spiel - University of Vienna
- Chetwyn Chan - The Hong Kong Polytechnic University
- Norbert Palz - Universität der Künste Berlin
- Wyn Morgan - University of Sheffield
- Thomas Hidber - University of Zürich
- Marilia de Nazare de Oliveira Ferreira - Federal University of Para
- Adam Habib - University of Witwatersrand
- Jan Gulliksen - KTH Royal institute of Technology
- Mwenda Ntarangwi - Commission for University Education in Kenya
- Bernd Engler - Universität Tübingen
- Folasade Ogunsola - University of Lagos
- Christina Maria Schönleber - Association of Pacific Rim Universities
- Jan Wöpking - German U15
- Sylvia Schwaag Serger - Lund University
- Katrin Dircksen - European Consortium for Innovative Universities
- Frank Coton - University of Glasgow
- Irvy Gledhill - University of Witwatersrand
- Philippa Pattison - University of Sydney
- Ada Pellert - FernUniversität in Hagen
- Elijah Bitange Ndemo - University of Nairobi
- Helene Dejoux - Agence Universitaire de la Francophonie
- Lee Kooi Cheng - National University of Singapore
- Eng Chye Tan - National University of Singapore
- Johan Geertsema - National University of Singapore
- Adrian Lee - National University of Singapore
- Heinz W. Engl - University of Vienna
- Carmen Zahn - University of Applied Sciences and Arts Northwestern Switzerland
- Santiba Campbell - Bennett College
- Georg Krausch - Johannes Gutenberg-Universität Mainz
- Keith B. Jenkins - Rochester Institute of Technology
- Lis Lange - University of Cape Town
- Volker Meyer-Guckel - Stifterverband
- Dercio Luiz Reis - Universidade Federal do Amazonas
- Ehab Abdel-Rahman - The American University in Cairo
- Sari Lindblom - University of Helsinki
- Ken Oyama - Universidad Nacional Autónoma de México
- Cristiane Derani - Universidade Federal de Santa Catarina
- Melchor Sanchez - National Autonomous University of Mexico
- Sebastian Kubis - FernUniversität in Hagen
- Dalmo Mandelli - Federal University of ABC (UFABC)
- John Okumu - Kenyatta University
- Joshua G. Bagaka’s - KCA University
- Bente Merete Stallknecht - University of Copenhagen
- Folasade Ogunsola, University of Lagos

1. Types of institutions overlap to some extent as participants could select from several characteristics to describe their institutions. [↑](#footnote-ref-1)
